# AAV2-VEGF-B gene therapy failed to induce angiogenesis in ischemic porcine myocardium due to inflammatory responses

**DOI:** 10.1038/s41434-022-00322-9

**Published:** 2022-02-07

**Authors:** Henna Korpela, Jaakko Lampela, Jonna Airaksinen, Niko Järveläinen, Satu Siimes, Kaisa Valli, Tiina Nieminen, Minttu Turunen, Maria Grönman, Antti Saraste, Juhani Knuuti, Mikko Hakulinen, Pekka Poutiainen, Vesa Kärjä, Jussi Nurro, Seppo Ylä-Herttuala

**Affiliations:** 1 A.I.Virtanen Institute for Molecular Sciences, University of Eastern Finland, Kuopio, Finland; 2 Kuopio Center for Gene and Cell Therapy, Kuopio, Finland; 3 Turku PET Centre, University of Turku, Turku, Finland; 4 Kuopio University Hospital, Kuopio, Finland; 5 Heart Center and Gene Therapy Unit, Kuopio University Hospital, Kuopio, Finland

**Keywords:** Gene delivery, Imaging, Gene therapy

## Abstract

Therapeutic angiogenesis induced by gene therapy is a promising approach to treat patients suffering from severe coronary artery disease. In small experimental animals, adeno-associated viruses (AAVs) have shown good transduction efficacy and long-term transgene expression in heart muscle and other tissues. However, it has been difficult to achieve cardiac-specific angiogenic effects with AAV vectors. We tested the hypothesis whether AAV2 gene transfer (1 × 10^13^ vg) of vascular endothelial growth factor B (VEGF-B186) together with immunosuppressive corticosteroid treatment can induce long-term cardiac-specific therapeutic effects in the porcine ischemic heart. Gene transfers were delivered percutaneously using direct intramyocardial injections, improving targeting and avoiding direct contact with blood, thus reducing the likelihood of immediate immune reactions. After 1- and 6-month time points, the capillary area was analyzed, myocardial perfusion reserve (MPR) was measured with radiowater positron emission tomography ([^15^O]H_2_O-PET), and fluorodeoxyglucose ([^18^F]FDG) uptake was used to evaluate myocardial viability. Clinical chemistry and immune responses were analyzed using standard methods. After 1- and 6-month follow-up, AAV2-VEGF-B186 gene transfer failed to induce angiogenesis and improve myocardial perfusion and viability. Here, we show that inflammatory responses attenuated the therapeutic effect of AAV2 gene transfer by significantly reducing successful transduction and long-term gene expression despite the efforts to reduce the likelihood of immune reactions and the use of targeted local gene transfer methods.

## Introduction

There is a significant unmet clinical need for new therapeutic approaches for severe coronary artery disease, the so-called refractory angina. Therapeutic angiogenesis based on gene therapy could offer a novel treatment for this patient group [1]. The effectiveness of gene therapy depends strongly on the delivery method and viral vector used. Adeno-associated viruses (AAVs) have attracted interest in the field of myocardial gene therapy since they offer more stable and long-lasting transgene expression than the most commonly used adenoviruses and have the ability to transduce nondividing cells such as cardiomyocytes [2]. AAV2 based gene therapy has been studied in patients suffering from several diseases, such as lipoprotein lipase deficiency, neurological diseases, retinal diseases, and hemophilia [3–5]. AAV2 has previously been shown to efficiently transduce mouse myocardium with lesser myocardial damage when compared to AAV9 (ref. [6].

Vascular endothelial factors are the most widely studied growth factors to induce therapeutic angiogenesis in the heart [7]. A member of this growth factor family, vascular endothelial growth factor B (VEGF-B186), has been shown to induce myocardium-specific angiogenesis and to improve myocardial perfusion both under normoxic and ischemic conditions [8, 9]. Besides angiogenic effects, VEGF-B also has a protective role in cardiomyocyte metabolism under ischemia [10].

Different AAV serotypes have been widely tested in mice showing no cytotoxicity or significant inflammatory responses [6, 11, 12]. However, in human clinical trials using systemic delivery of AAV vectors, the most concerning issue has been the high prevalence of preexisting antibodies and immune responses against the virus leading to a reduced therapeutic effect [13–17]. It has been observed that the host immune response to AAV capsid is primarily mediated by circulating neutralizing antibodies (nAbs), and even low levels of nAbs can halt the transduction and long-term gene expression [14]. This subject is crucial since 30 to 80% of different human populations are positive for AAV2 neutralizing antibodies [18]. Regarding AAV serotype 2, Calcedo et al. have previously shown that the prevalence of nAbs to AAV2 is the highest among different AAV serotypes independent of the global region [19]. This is also a matter for translational research since seroprevalence of over 30% for anti-AAV2 nAbs has been observed in pigs [20].

This study evaluated the safety and efficacy of AAV2-VEGF-B gene therapy in a large animal model for chronic myocardial ischemia. As a control group, AAV2 encoding green fluorescent protein (GFP) was used. Myocardial ischemia was induced 14 days prior to the gene transfer, and the animals were followed up until 1- and 6-month time points. To prevent immune responses towards the viral vector, long-term peroral prednisolone therapy was used. Clinically relevant imaging modalities, such as ^15^O-radiowater positron emission tomography ([^15^O]H_2_O-PET) and radiolabeled fluorodeoxyglucose ([^18^F]FDG) PET, were used to evaluate the myocardial perfusion and viability. However, no long-term benefits were found due to immune responses against the AAV2 vector in spite of the local delivery and simultaneous corticosteroid treatment.

## Materials and methods

**Fig. 1:**
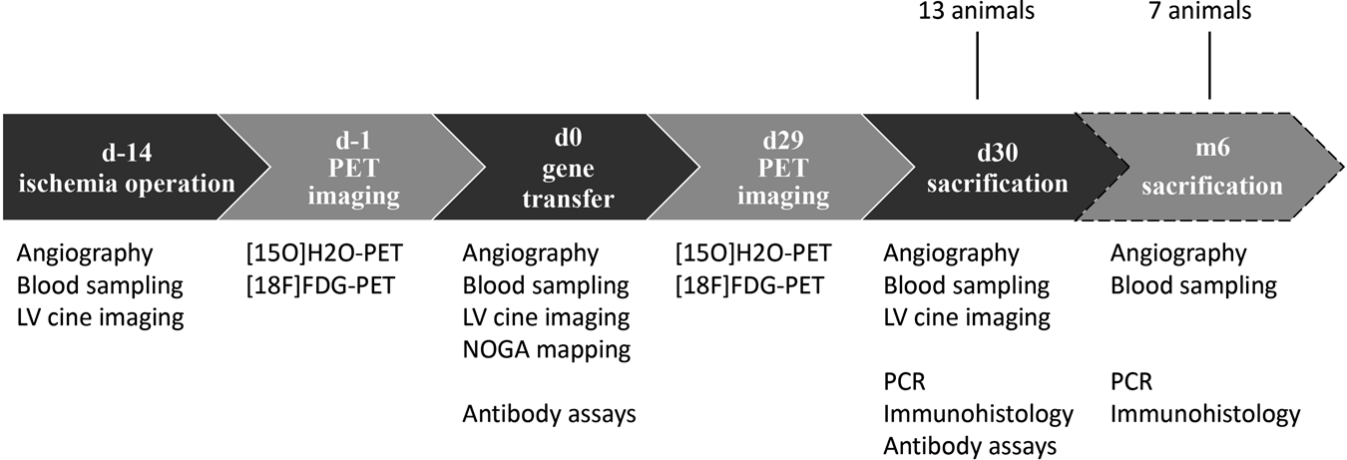
Study protocol. Thirteen animals were followed up until 1-month time point, and seven animals until 6-month time point. PET imaging was performed one day prior to the gene transfer and sacrification. Angiographic measurements and blood sampling were performed at the time of angiographic procedures and sacrification.

Pigs (*n* = 14) were randomly divided into three groups receiving 1 × 10^13^ vg of either AAV2-VEGF-B186 (*n* = 7), or AAV2-GFP (*n* = 7). Pigs were followed up until 1 month. One AAV2-VEGF-B186 pig did not reach this time point since it died prematurely. Another group of animals (*n* = 7) treated with 1 × 10^13^ vg of AAV2-VEGF-B186 (*n* = 3) or AAV2-GFP (*n* = 4) was followed up until 6-month time point. The study protocol is detailed in Fig. 1. All experiments were performed using female domestic pigs and were approved by the Animal Experiment Board in Finland. The animals were 3 months old and weighed ~30 kg at the beginning of the experiments. Sample size was determined by using resource equation method. The animals were randomized into the groups before the gene transfer and the investigators remained blinded until the data was analyzed.

### AAV2 vectors

AAV2 vectors were prepared by National Virus Vector Laboratory and Kuopio Center for Gene and Cell Therapy. Briefly, the production of the vectors was based on HEK293 cell transfection using pAAV2 vector plasmids and pDG2 helper plasmid (Plasmid Factory) complexed with polyethyleneimine (PEIpro, Polyplus transfection). Affinity chromatography was used for AAV2 vector purification [21]. AAV2 preparations were tested for sterility, mycoplasma, infectivity, and functionality.

### Bottleneck stent model

To induce chronic myocardial ischemia, a bottleneck stent was placed in the proximal part of the anterior descending branch of the left coronary artery 14 days before the gene transfer, as described in our previous studies [22].

### Medication

Animals received daily 200 mg of amiodarone and 2.5 mg of bisoprolol perorally to prevent fatal ventricular arrhythmias. The medication started 1 week before the ischemia operation and continued daily until the end of the follow-up. Loading doses of clopidogrel (300 mg, peroral) and acetylsalicylic acid (300 mg, peroral) were administered 1 day before the ischemia operation to prevent in-stent thrombosis after the bottleneck stent placement. Also, 30 mg enoxaparin was administered intravenously at the beginning of the ischemia operations and subcutaneously after the operation. To prevent ventricular arrhythmias during the ischemia operation, 100 mg of lidocaine and 2.5 mL MgSO_4_ were administered intravenously. As infection prophylaxis, cefuroxime (500 mg, intramuscular) was administered at the beginning of each operation.

Before the operations, pigs were sedated with an intramuscular injection of 1.5 mL atropine and 6 mL of azaperone. After the initial sedation, animals were put under general propofol and fentanyl anesthesia with doses of 15 mg/kg/h and 10 µg/kg/h.

To reduce inflammatory responses to the vector, a daily peroral 40 mg dose of prednisolone was given to all animals for 3 weeks, beginning 3 days before the gene transfer procedure. The treatment was then continued with 20 mg prednisolone daily. Simultaneously, 40 mg of peroral pantoprazole was administered daily to prevent possible ulcus formation caused by the high prednisolone dose.

### Cardiac output measurement

Cardiac output (l/min) was measured by left ventricular cine imaging using fluoroscopic imaging at rest and under dobutamine-induced stress at increasing infusion rates. Upon reaching the target heart rate of 160 bpm, the infusion rate was kept constant during fluoroscopic imaging. The cardiac output was calculated by using the software of the angiographic station (GE Innova 3100IQ Pro, NY, USA) and Simpson’s rule. Cardiac output was then normalized to the weight of the animal at the moment of imaging.

### Gene transfer

For the gene transfer procedures, an intramyocardial injection catheter, MyoStar^®^ (Biosense Webster, a Johnson & Johnson company, Diamond Bar, CA, USA), was introduced to the left ventricle via a femoral sheath. Under fluoroscopic guidance (GE Innova 3100IQ Pro, NY, USA) and using the NOGA^®^ mapping system (Biologics Delivery Systems, a Johnson & Johnson company, Irwindale, CA, USA), an electroanatomical map of the left ventricle was acquired. Using this map as a guide, 1 × 10^13^ vg divided into ten injections (300 μl each) were injected into the hypokinetic but still viable areas of the left ventricle. For viability, a unipolar voltage over 5 mV was used as a criterion. For hypokinesia, a local linear shortening (LLS) at least below 12% but preferably below 6% was selected [23]. The injection needle length was set for 3 mm. An injection duration was 30 s, and the injection needle was kept inside the myocardium for an additional five seconds before retraction to prevent backflow into the ventricle.

### Sacrification

Animals were sacrificed with intravenous KCl injection under general anesthesia. The heart was perfused with PBS (Dulbecco’s Phosphate Buffered Saline). Tissue samples were collected from the gene transfer site in the heart, and safety tissue samples were collected from lung, liver, spleen, kidney, ovary, brain, retina, and both proximal (thoracic cavity) and distal (femoral plexus) lymph nodes. Samples were fixed in 4% paraformaldehyde (PFA, pH 7.2) for 48 h at 4 °C and then placed in 15% sucrose for at least 48 h before embedding.

### Capillary analysis

Mean capillary area (%) was measured from CD31-immunostained (1:100, AF806; R&D) sections of the pig myocardium [9]. All measurements were done with Fiji software in a blinded manner from 25 randomly selected fields at ×200 magnification from five sections of each pig.

### Transgene mRNA expression and AAV2-vector biodistribution

To evaluate transgene mRNA expression in the myocardium, three samples from the gene transfer area from four VEGF-B186 and four GFP animals were analyzed, in addition to control samples from the posterior wall of the left ventricle. Half of the analyzed animals from both groups were sacrificed at a 1-month time point and the rest at a 6-month time point. Also, transgene mRNA was quantified from the extracardiac samples of the four animals from the VEGF-B186 group. VEGF-B186 group was chosen since the higher mRNA expressions in the heart.

Tissue RNA was extracted using TRI Reagent^®^ (Life Technologies) and treated with DNAse (DNA-Free™, Life Technologies), according to the manufacturer’s instructions. cDNA was synthesized from 1 µg of total RNA using RevertAid Reverse Transcriptase (Thermo Scientific) and Random Hexamer Primer (Thermo Scientific), and the levels of VEGF-B186 and GFP cDNA were measured by qPCR using VEGF-B186 and GFP specific Taqman-based assays (Integrated DNA Technologies). The expression of VEGF-B186 and GFP in the samples was normalized to the expression of the housekeeping gene HPRT (Applied Biosystems; ss03388274 m1 HPRT1). Samples were considered negative for transgene mRNA expression if no amplification was seen after 40 cycles.

To study the biodistribution of the AAV2 vector, we analyzed the vector genome copy numbers from different tissues. The tissue DNA was extracted using NucleoSpin^®^ DNA RapidLyse kit (Machery-Nagel), according to the manufacturer’s instructions. The total amount of AAV2 vector was quantitated using AAV2 ITR specific PrimeTime^®^ qPCR assay (Integrated DNA Technologies) and TaqMan Fast Advanced Master Mix (Applied Biosystems) and measured with StepOnePlus™ Real-Time PCR instrument (Applied Biosystems). Samples were considered negative for AAV2 ITR if no amplification was seen after 40 cycles.

### Radiowater perfusion imaging

Rest and stress [^15^O]H_2_O PET/CT scans were performed using a Siemens Biograph mCT scanner (Siemens Healthcare, Erlangen, Germany). Computed tomography (CT) scans were performed at rest, and stress imaging and CT information were used for attenuation correction. An on-site cyclotron (PETtrace 860, GE Healthcare, UK) and radiowater generator (Hidex Oy, Finland) produced [^15^O]H_2_O bolus. Rest and stress imaging was performed using an 800 MBq [^15^O]H_2_O bolus. The dynamic acquisition included frames of 14 × 5, 3 × 10, 3 × 20, and 4 × 30 s (total duration 280 s). After suitable decay of 12 min, stress imaging was performed with a further 800 MBq [^15^O]H_2_O bolus. The dynamic acquisition was performed during adenosine-induced hyperemia. Images were reconstructed on a 128 × 128 matrix using the ordered subsets expectation maximization (OSEM) iterative algorithm (2 iterations, 21 subsets, zoom 2, Gaussian 6 mm post-filter).

### Myocardial perfusion reserve analysis

Regional myocardial perfusion (mL/g/min) was measured using Carimas 2 software (Turku PET Center, Turku, Finland; http://www.turkupetcentre.fi/carimas). Gene transfer area was selected as a region of interest (ROI) by comparing the PET image to the NOGA map from the ischemia operation [24]. The blood perfusion of the gene transfer area was normalized to the area of maximal perfusion of each heart both at rest and at stress. Myocardial perfusion reserve (MPR) was calculated as the ratio of the perfusion at stress to rest.

### [^18^F]FDG-PET imaging

The pigs received intravenous [^18^F]FDG injections of 363 ± 15 MBq. To induce euglycemic hyperinsulinemic clamp, intravenous boluses of insulin (10 IU) and glucose (1 g/kg) were administered to the animals. Imaging was performed 60 min after [^18^F]FDG injection with the Siemens Biograph mCT PET/CT scanner (Siemens Healthcare, Erlangen, Germany). CT scan was used for attenuation correction. PET acquisition was performed in one bed position using an acquisition time of 10 min. The images were reconstructed with ordered subset expectation maximization (OSEM) algorithm using 2 iterations and 21 subsets (matrix size was 256 × 256, Gaussian 3 mm post-filter).

Quantification of the [^18^F]FDG uptake in the myocardium was performed as previously described [25]. Briefly, the myocardial contours from the [^15^O]water images were copied to the co-registered [^18^F]FDG images. The polar maps of [^18^F]FDG uptake expressed as standardized uptake value (SUV) in the LV myocardium were generated using matching image orientation and sampling points. ROI defining the ischemic area was copied from the [^15^O]water polar maps to measure [^18^F]FDG uptake in this region. The mean SUVs were determined from the gene therapy area and the ischemic area defined as resting myocardial blood flow <67% of the remote area [26]. The apical segment 17 was excluded from the analysis.

### Detection of anti-AAV2 IgG antibodies

Serum anti-AAV2 antibodies were quantified by enzyme-linked immunosorbent assay (ELISA). Recombinant AAV2 particles were diluted in PBS (pH 7.4), and 100 μl was added to the wells in a 96-well Nunc MaxiSorp plate (Thermo Fisher Scientific, Waltham, USA) and incubated at 4 °C overnight. PBS without AAV2 was used as a no coating control. On the following day, the plate was washed four times with PBS, and 150 μl of blocking solution (5% BSA in PBS) was added to each well and incubated at 37 °C for 2 h. The plate was again washed four times with PBS, and 100 μl of pig serum diluted from 1:100 to 1:24300 was added and incubated at 37 °C for 90 min. After four washes, 100 μl of anti-pig IgG peroxidase antibody (Sigma-Aldrich, St. Louis, USA) was added and incubated at 37 °C for 1 h. After washing, 100 μl of tetramethylbenzidine (TMB) substrate solution (Sigma-Aldrich) was added and incubated for 20 min at room temperature. 100 μl of Stop reagent for TMB substrate (Sigma-Aldrich) was added, and absorbances were read at 450 and 650 nm.

### Neutralizing anti-AAV2 antibody assay

On day 1, 96-well plates were seeded with 1.5 × 10^4^ 293T cells per well. On day 2, Compound C (Sigma-Aldrich) (ref. [27]) was added to the wells in a final concentration of 5 µM in serum-free DMEM and incubated for 1 h at 37 °C and 5 % CO_2_. Serum samples were heat-inactivated at 56 °C for 30 min. Serum, subjected to threefold serial dilutions from 1:3 to 1:19683 with fetal bovine serum, was incubated with AAV2 expressing murine secreted alkaline phosphatase (muSEAP) at 37 °C for 1 h; this mixture was then added to a culture well pre-treated with Compound C, and incubated at 37 °C and 5% CO_2_ for 24 h. Pig serum dilution series without AAV2-muSEAP was used as a negative control as well as DMEM only, and AAV2-muSEAP without the serum was used as a positive control. On day 3, the culture medium was collected, and muSEAP expression was measured with a Phospha-Light chemiluminescent reporter gene assay system (Applied Biosystems, Foster City, USA). The neutralizing titer was reported as the highest serum dilution that inhibited the AAV2-muSEAP transduction by ≥50% compared with the positive control without serum.

### Isolation of peripheral blood mononuclear cells

To investigate a possible T-cell response towards AAV2, four additional animals received an intramyocardial AAV2 gene transfer, and fresh blood samples were collected before and 1 month after the gene transfer. Peripheral blood mononuclear cells (PBMC) were isolated from blood samples by using Ficoll-Paque PLUS (Sigma-Aldrich) and SepMate tubes (STEMCELL, Vancouver, Canada) by the instructions of the manufacturer. Isolated PBMC were resuspended in RPMI-1640 media supplemented with 10% fetal bovine serum (FBS) and 2 mM L-glutamine.

### IFNgamma ELISpot assay

The number of AAV2-specific interferon-gamma-producing PBMC was determined using Porcine IFNgamma ELISpot kit (R&D). Briefly, 200,000 fresh isolated mononuclear cells were seeded per well and incubated with AAV2-mock (1.05 × 10^11^ vg/ml or 0.50 × 10^11^ vg/ml) at 37 °C and 5% CO_2_ for 48 h. Unstimulated PBMC were used as a negative control as well as cell cultures with media only. PBMC stimulated with phytohemagglutinin (PHA) were used as a positive control. Spots were counted manually by an optical microscope.

### Statistical analysis

Unpaired *t* test was used for statistical analysis of capillary analysis data, [^15^O]H_2_O-PET, and [^18^F]FDG-PET data. The cardiac output data were analyzed by using ANOVA following the Bonferroni post-test.

## Results

### No angiogenesis or change in myocardial perfusion reserve was seen after AAV2-VEGF-B186 gene transfer

**Fig. 2:**
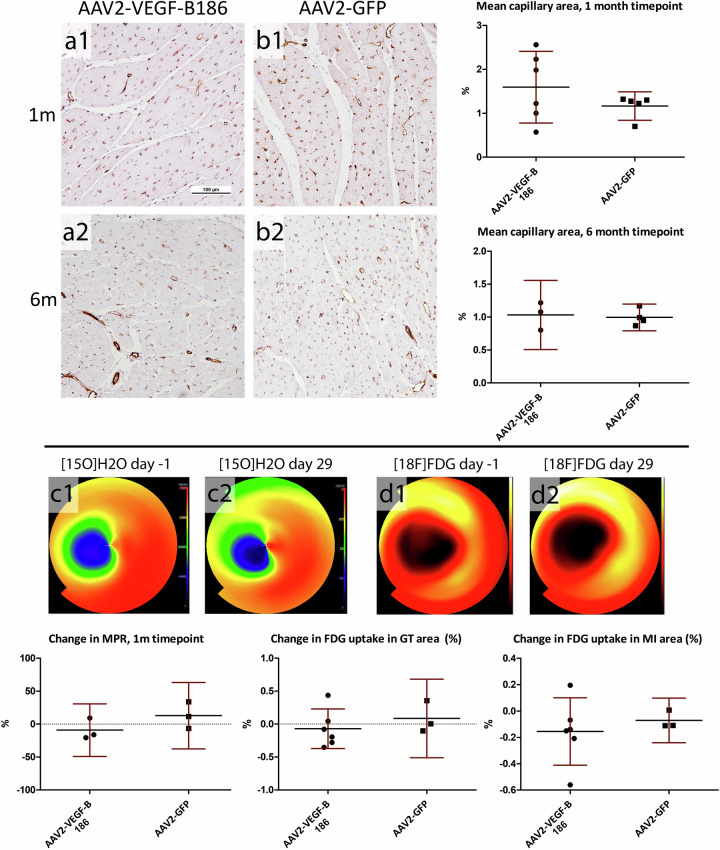
No angiogenesis in AAV2-VEGF-B186 transduced hearts was observed. The mean capillary area was at the same level in the (a1–a2) AAV2-VEGF-B186 hearts compared to (b1–b2) the control group. c1–c2 There was no improvement in myocardial perfusion reserve measured by [^15^O]H_2_O-PET. d1–d2 Analysis of [^18^F]FDG-PET imaging showed no change in FDG uptake at the gene transfer or the infarction area. Results are represented as dot plots separately for each individual animal and as mean ± 95% CI. Scale bar: 100 µm.

**Fig. 3:**
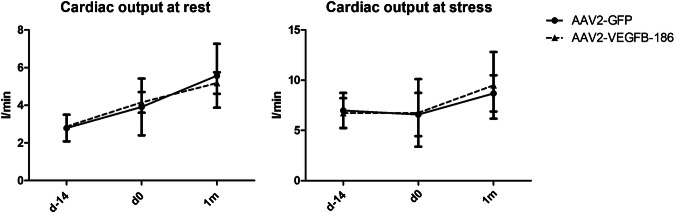
No improvement in cardiac output was seen after 1-month follow-up. Lines represent the group mean ± 95% CI.

AAV2-VEGF-B186 gene transfer did not increase the mean capillary area measured from the myocardial sections compared to AAV2-GFP-transduced hearts after 1- and 6-month follow-up. AAV2-VEGF-B186 gene transfer did not increase MPR at the gene transfer area as measured by [^15^O]H_2_O-PET after 1-month follow-up. Uptake of FDG in the gene transfer area or at the infarction area was at the same level in the VEGF-B186 transduced hearts as in the GFP-transduced hearts, (Fig. 2). Also, no improvement in cardiac function was seen 1 month after the gene transger (Fig. 3).

### AAV2 genome copies were low in the myocardium 1 month and 6 months after the gene transfer

**Fig. 4:**
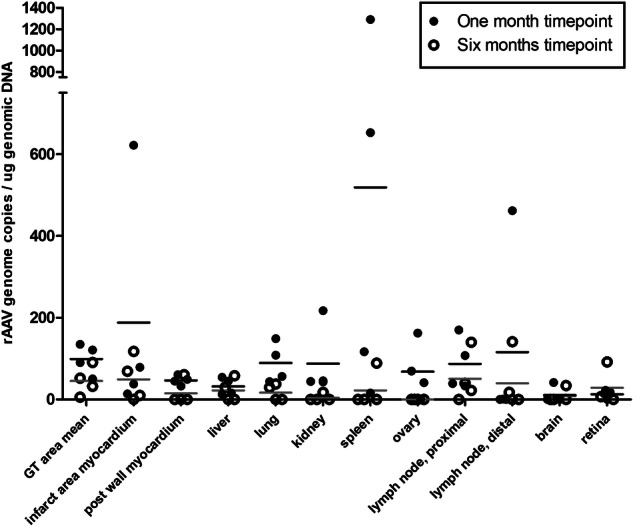
AAV2-vector genome copy numbers remained low in all collected tissues, including the myocardial samples from the gene transfer and infarct areas. AAV2 GT area mean consists of ten independent samples from the target area of each myocardium. Results are expressed as a dot plot, each dot representing an individual animal and as a mean.

The vector distribution and transgene expression were evaluated by PCR quantification of AAV ITR in four randomly chosen animals from both groups sacrificed at 1-month time point, and four animals from both groups sacrificed at 6-month time point. Analysis revealed a very low amount of vector genome copies in all the collected samples, including the samples from the gene transfer area (Fig. 4). The highest genome copy numbers were seen in the spleen samples.

### Transgene mRNA was expressed in the gene transfer area

RT-PCR-mediated relative quantification showed transgenic mRNA expression in the gene transfer area in animals receiving AAV2-VEGF-B186 and AAV2-GFP at 1-month and at 6-month time points. Of the analyzed animals, only one was observed to express the transgene in the intracardiac control tissue from the posterior wall of the left ventricle (Table 1). Extracardiac samples of four AAV2-VEGF-B186 transduced animals were analyzed for transgene mRNA expression by RT-PCR. VEGF-B186 mRNA could be found only from one liver sample from one animal. All other extracardiac samples remained negative for VEGF-B186 mRNA.

**Table 1:** Transgene mRNA expression was found in the myocardium of all AAV2-VEGF-B186 transduced animals and three AAV2-GFP-transduced animals.

		GFP/HPRT		VEGF-B186/HPRT						
	Transgene	GT area mean	Post. wall	GT area mean	Post. wall	Lung	Liver	Spleen	Kidney	Proximal lymph node
Pig 1	VEGF-B186	ND	ND	110.7	1.1	ND	1.0	ND	ND	ND
Pig 2		ND	ND	27	ND	ND	ND	ND	ND	ND
Pig 3		ND	ND	26.9	ND	ND	ND	ND	ND	ND
Pig 4		ND	ND	66.9	ND	ND	ND	ND	ND	ND
Pig 5	GFP	ND	ND	ND	ND					
Pig 6		5.0	ND	ND	ND					
Pig 7		2.0	ND	ND	ND					
Pig 8		16.9	ND	ND	ND					

Gene therapy (GT) area mean consists of three independent samples from each myocardium. Results are presented as the quantity of the transgenic mRNA divided by the quantity of the HPRT mRNA.

*ND* not detected.

### Serum anti-AAV2 IgG and neutralizing antibodies were detected in high quantities

**Fig. 5:**
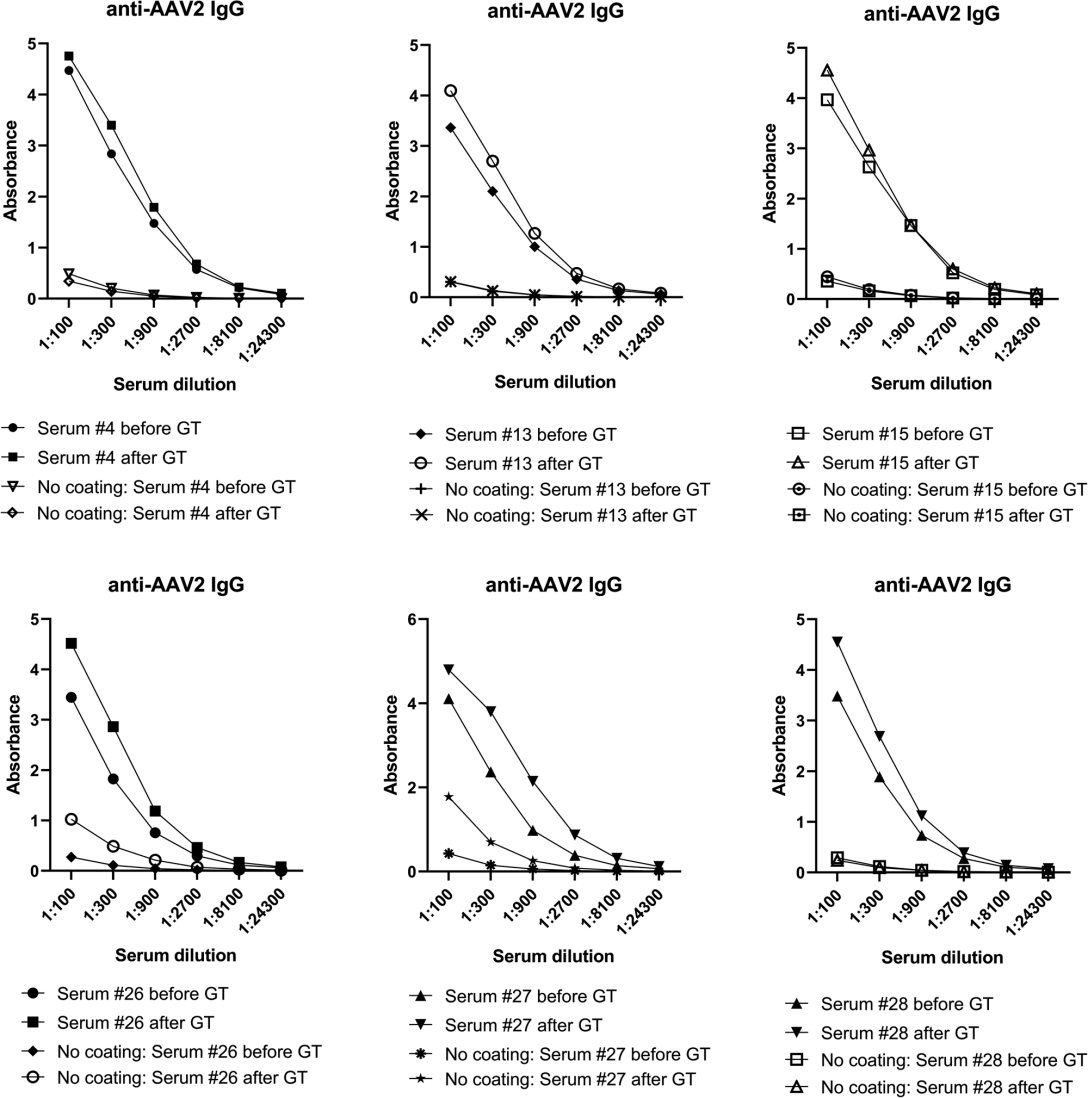
High levels of anti-AAV2 IgG antibodies were detected in the pig sera. Binding anti-AAV2 IgGs were measured from the sera of six animals with a solid-phase ELISA assay before and 1 month after the gene transfers. Each graph represents an individual animal.

**Fig. 6:**
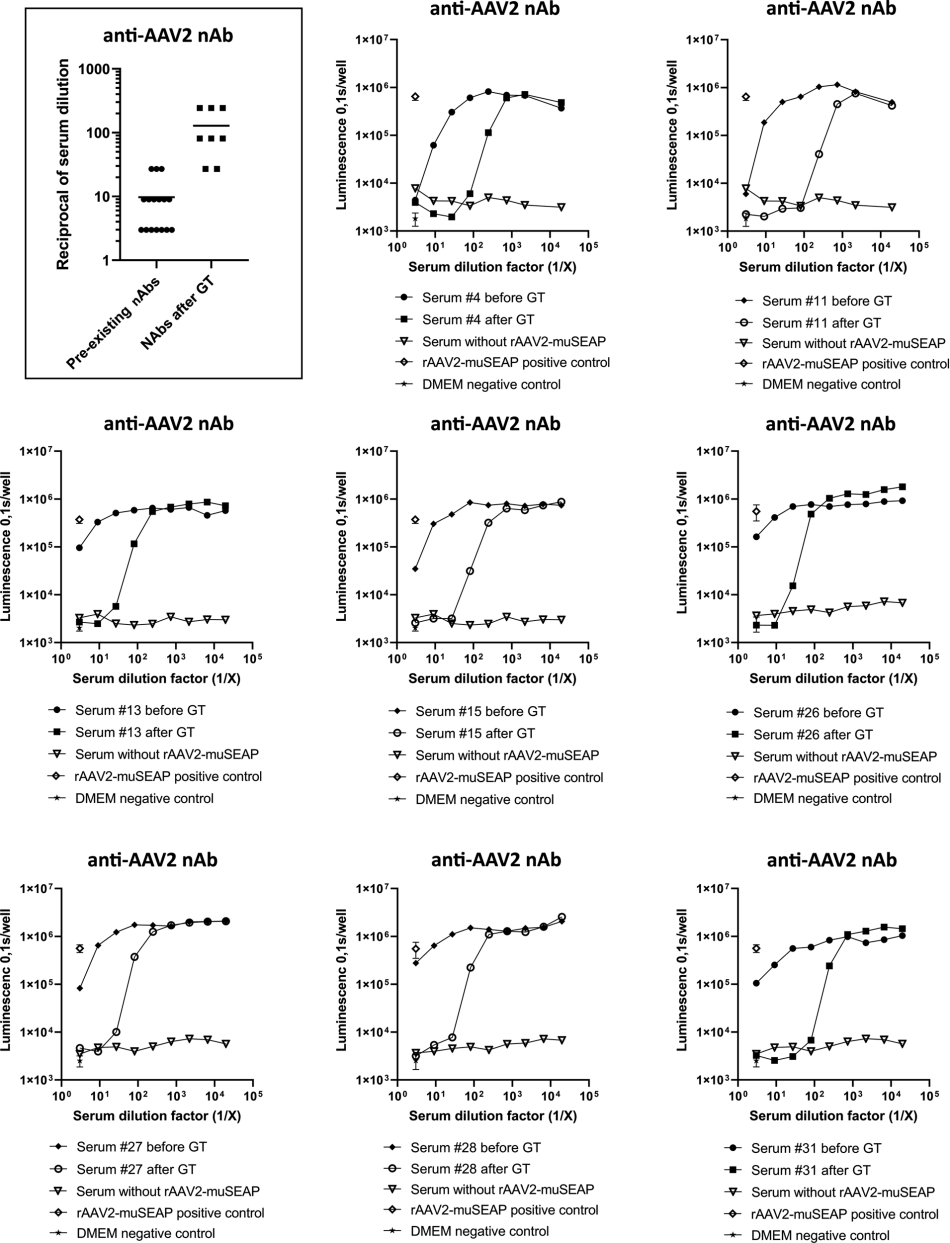
Neutralizing antibodies in the pig sera. nAbs were measured from eight animals before and after the AAV2 gene transfer. Each graph represents an individual animal. The curves present the capability of the serum anti-AAV2 antibodies to prevent AAV2-muSEAP transduction in 293T cells. The framed combined dot plot shows the serum dilution needed to inhibit AAV2-muSEAP transduction efficacy of more than 50%.

All animals tested for anti-AAV2 IgG antibodies showed high antibody levels after the gene transfers (Fig. 5). Detectable antibody levels were observed even at 1:900 serum dilution and in some animals at 1:2700 dilution. Levels of IgG antibodies increased after the gene transfer, suggesting a humoral immune response towards the AAV2. The anti-AAV2 nAb levels rose in all animals after the AAV2 gene transfer (*n* = 8), and serum dilutions as high as 1:243 were shown to block the transduction (Fig. 6).

### T cells were observed at the gene transfer area

**Fig. 7:**
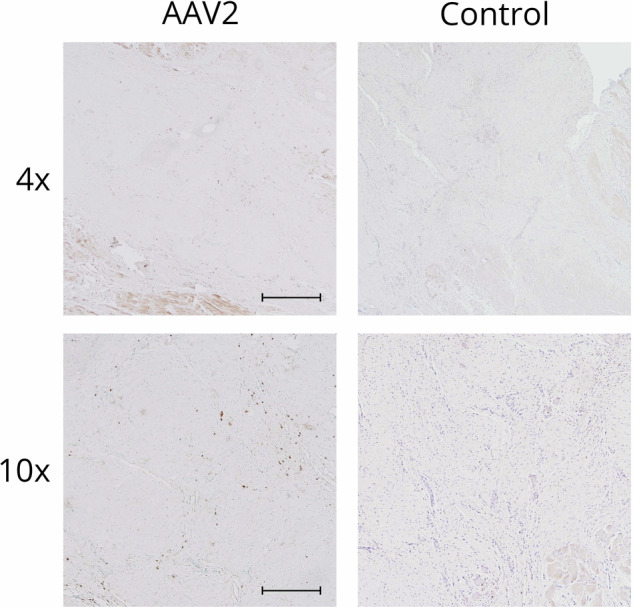
CD3 immunostaining of myocardial sections. Several CD3 positive cells were observed at the gene transfer area of AAV2-transduced animals at 1-month time point, whereas only individual CD3 positive cells were seen in the control samples collected from ischemic animals that had not gone through any gene transfer. Scale bar in 4×: 500 µm. Scale bar in 10×: 100 µm.

Multiple T cells were recognized by CD3 immunostaining from the gene transfer area of AAV2-transduced hearts at 1 month after the gene transfer. Only individual CD3 positive cells were observed in non-transduced hearts with similar ischemia (Fig. 7).

### Inflammatory response was seen in lymphatic tissues

**Fig. 8:**
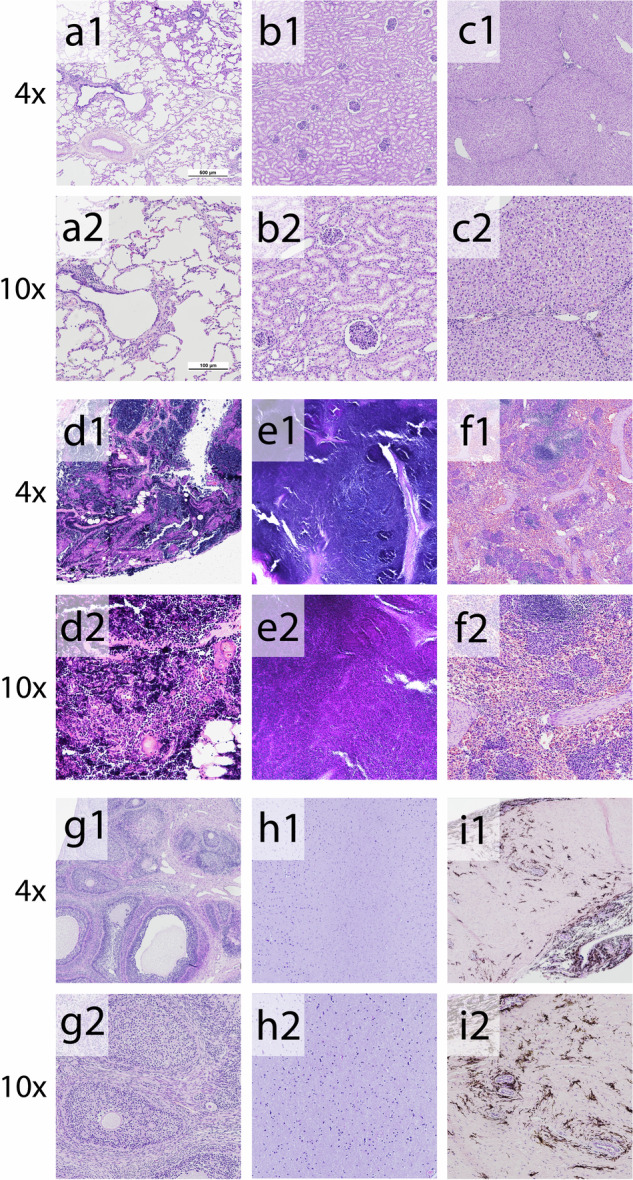
Histology of selected organs and tissues. Hematoxylin–eosin-stained tissues collected from AAV2-transduced animals showed normal histology except in lymph nodes. Both (d1–d2) proximal lymph nodes collected from the thoracic cavity and (e1–e2) distal lymph nodes from the groin showed robust reactivity suggesting a systemic inflammatory response to the AAV2 gene transfer vector. Magnification: ×40 and ×100. a1–a2 lung (b1–b2) kidney (c1–c2) liver, (d1–d2) lymph node proximal (e1–e2) lymph node distal (f1–f2) spleen, (g1–g2) ovario, (h1–h2) brain, (i1–i2) retina.

Examination of the samples collected from other tissues—lung, liver, spleen, kidney, ovary, brain, retina—showed normal histology. However, consistent with immune activation, both in proximal thoracic and distal groin lymph nodes, a robust reactivity was observed (Fig. 8).

### No specific T-cell response was detected by ELISpot

No specific T-cell response towards the AAV2 vector was detected in ELISpot before or 1 month after the intramyocardial AAV2 gene transfer since the isolated PBMC stimulated two either concentrations of AAV2 vector did not secrete IFNgamma (data not shown).

### Clinical chemistry parameters remained unchanged during the follow-up

Blood samples were collected before and 30 days after the gene transfer. Laboratory parameters measured from the pig sera remained unchanged during the follow-up (Table 2).

**Table 2:** Clinical chemistry (mean ± SD).

	1-month follow-up				6-month follow-up			
Group	AAV2-VEGF-B186 (*n* = 7)		AAV2-GFP/LacZ (*n* = 7)		AAV2-VEGF-B186 (*n* = 3)		AAV2-GFP/LacZ (*n* = 4)	
Time point	Pre-GT	1 Month	Pre-GT	1 Month	Pre-GT	1 Month	Pre-GT	1 Month
TnI (ng/mL)	0.50 ± 0.08	0.16 ± 0.09	0.52 ± 0.10	0.08 ± 0.03	0.35 ± 0.09	0.34 ± 0.16	3.56 ± 3.01	0.13 ± 0.04
ALAT (U/L)	79 ± 4	58 ± 5	75 ± 6	65 ± 8	72 ± 8	74 ± 7	83 ± 10	73 ± 5
AFOS (U/L)	148 ± 10	153 ± 19	168 ± 22	181 ± 21	157 ± 22	110 ± 10	157 ± 13	103 ± 10
CRP (mg/L)	46 ± 10	36 ± 10	52 ± 21	42 ± 5	37 ± 9	27 ± 8	30 ± 14	19 ± 8
Creatinine (µmol/L)	103 ± 5	118 ± 7	106 ± 4	124 ± 7	110 ± 7	165 ± 7	92 ± 3	167 ± 3

## Discussion

Previous studies with adenoviral VEGF-B186 gene transfer have shown significant angiogenic effects and improved myocardial perfusion with similar delivery methods and imaging modalities [8, 9]. However, no angiogenesis or improvement of myocardial perfusion or viability was seen after the AAV2-VEGF-B186 gene transfer in this study.

Transgene expression was detectable in the myocardium, but only a low amount of AAV2-vector genome was detected in the gene transfer area at 1-month time point by PCR. The low level of the detected AAV2 genome is most likely due to the elimination of the viral particles by the immune system. During the follow-up, the AAV2 vectors still succeeded in transducing the host cells, leading to the low-level production of the transgene mRNA. However, the transgene expression was insufficient to induce significant angiogenesis and clinically relevant improvement in cardiac perfusion.

A significant increase in IgG and nAb levels was detected 1 month after the gene transfer indicating a specific humoral response towards the AAV2 capsid. In preclinical studies and clinical trials, it has been shown that nAb titers of 1:2 and 1:4 can inhibit successful transduction [13, 14, 28–31]. Furthermore, histological examination of the proximal and distal lymph nodes showed a robust reactivity, and T cells were seen at the gene transfer area 1 month after the gene transfer. Thus, we conclude that the potential effect of AAV2-VEGF-B186 gene therapy was halted by the humoral immune responses. Despite the strong humoral immune responses, the specific T-cell response towards AAV2 was not detected by IFNgamma ELISpot. However, the targeted delivery method and the long follow-up time could have reduced the measurable T-cell activation.

Previously it has been shown that AAV1 and AAV9 can successfully transduce pig myocardium [32–34]. However, the AAV2 vector has mostly been used in preclinical and clinical studies, but the presence of preexisting neutralizing antibodies has inhibited successful transduction and long-term therapeutic effect in humans after systemic delivery [14].

In this study, local intramyocardial delivery and long-term prednisolone treatment were used to suppress the immune responses toward the viral capsid [35]. However, this was not effective enough since elevations in IgG and nAb levels were seen despite the prednisolone treatment. In the future, a more potent immunosuppressant might be used to suppress the immune response towards the AAV2 capsid during and after the gene transfer. However, immunomodulatory drugs also pose a risk for opportunist infections and other complications. Also, transduction efficacy could be improved by using a higher dose of the viral vector or altering the vector serotype and screening for preexisting nAbs. However, it is plausible to conclude that immune responses will reduce the efficacy of intracardiac AAV2 gene therapy in human clinical trials unless the immunological reactions can be controlled.

## Limitations of the study

As typical for large animal studies, the number of animals was limited. Also the N for PCR qualification was small due to the need for analyzation of multiple tissue samples per heart demanded by the local needle injection delivery method. Also, in the future, antibody levels could be measured from multiple time points after the gene transfer to detect the exact timing of the humoral immune response.

## Data Availability

The datasets analysed during the current study are available from the corresponding author on reasonable request.
