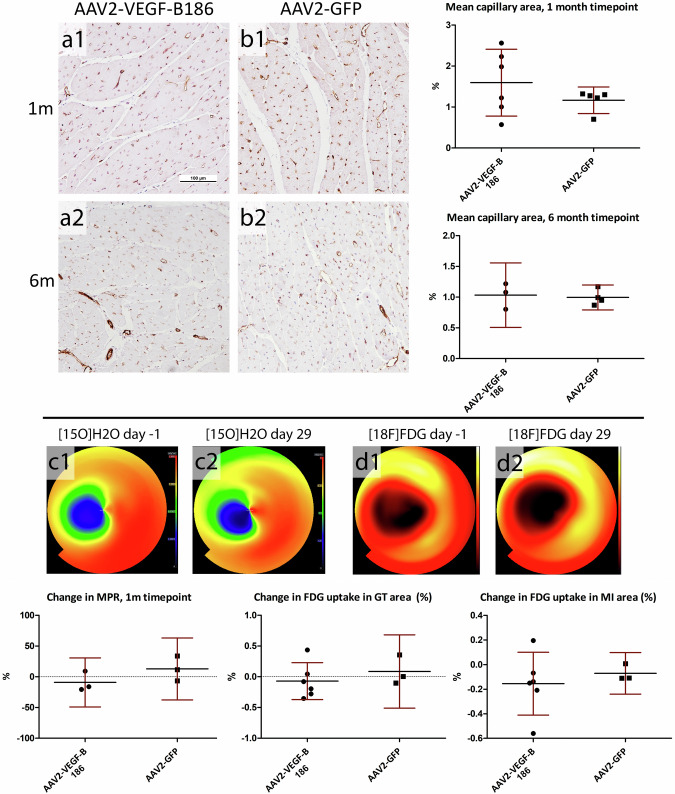# Correction: AAV2-VEGF-B gene therapy failed to induce angiogenesis in ischemic porcine myocardium due to inflammatory responses

**DOI:** 10.1038/s41434-024-00481-x

**Published:** 2024-08-26

**Authors:** Henna Korpela, Jaakko Lampela, Jonna Airaksinen, Niko Järveläinen, Satu Siimes, Kaisa Valli, Tiina Nieminen, Minttu Turunen, Maria Grönman, Antti Saraste, Juhani Knuuti, Mikko Hakulinen, Pekka Poutiainen, Vesa Kärjä, Jussi Nurro, Seppo Ylä-Herttuala

**Affiliations:** 1 A.I.Virtanen Institute for Molecular Sciences, University of Eastern Finland, Kuopio, Finland; 2 Kuopio Center for Gene and Cell Therapy, Kuopio, Finland; 3 Turku PET Centre, University of Turku, Turku, Finland; 4 Kuopio University Hospital, Kuopio, Finland; 5 Heart Center and Gene Therapy Unit, Kuopio University Hospital, Kuopio, Finland

**Keywords:** Gene delivery, Imaging, Gene therapy

Correction to: *Gene Therapy* 10.1038/s41434-022-00322-9, published online 7 February 2022

In the article “AAV2-VEGF-B gene therapy failed to induce angiogenesis in ischemic porcine myocardium due to inflammatory responses” by Korpela H, Lampela J, Airaksinen J, Järveläinen N, Siimes S, Valli K, Nieminen T, Turunen M, Grönman M, Saraste A, Knuuti J, Hakulinen M, Poutiainen P, Kärjä V, Nurro J, Ylä-Herttuala S. (Gene Therapy, 2022; Vol 29, 643-652, 10.1038/s41434-022-00322-9) there was an error in the Figure 2 considering the [18F]FDG reference image (d2) which was from d0, not d29 as intended. Figure 2 has now been corrected with image from d29 (d2).